# Rod and Cone Dark Adaptation in Congenital Aniridia and Its Association With Retinal Structure

**DOI:** 10.1167/iovs.64.4.18

**Published:** 2023-04-17

**Authors:** Hilde R. Pedersen, Stuart J. Gilson, Erlend C. S. Landsend, Øygunn A. Utheim, Tor P. Utheim, Rigmor C. Baraas

**Affiliations:** 1 National Centre for Optics, Vision and Eye Care, Faculty of Health and Social Sciences, University of South-Eastern Norway, Kongsberg, Norway; 2 Department of Ophthalmology, Oslo University Hospital, Oslo, Norway

**Keywords:** congenital aniridia, dark adaptation, retinal structure, optical coherence tomography

## Abstract

**Purpose:**

To characterize the association between dark-adapted rod and cone sensitivity and retinal structure in *PAX6*-related aniridia.

**Methods:**

Dark-adaptation curves were measured after a 5-minute exposure to bright light with red (625 nm) and green (527 nm) 2° circular light stimuli presented at ≈20° temporal retinal eccentricity in 27 participants with aniridia (nine males; 11–66 years old) and 38 age-matched healthy controls. A two-stage exponential model was fitted to each participant's responses to determine their cone and rod thresholds over time. The thicknesses of macular inner and outer retinal layers were obtained from optical coherence tomography images in 20 patients with aniridia and the 38 healthy controls. Aniridia-associated keratopathy (AAK) grade (0–3) and lens opacities were quantified by clinical examination of the anterior segment.

**Results:**

The rod–cone break time was similar between patients with aniridia and healthy controls. Dark-adapted cone and the rod thresholds were higher in aniridia compared with healthy controls. In aniridia, foveal outer retinal layer thickness correlated with both final cone and rod thresholds. A multiple regression model indicated that foveal outer retinal layer thickness and age were the main explanatory variables to predict both final cone and rod thresholds in aniridia when the AAK grade was 2 or less.

**Conclusions:**

The results show that both rod- and cone-related functions are affected in *PAX6-*related aniridia and suggest that retinal anatomical and physiological changes extend beyond the area commonly studied in this condition: the central macula.

Aniridia is a rare genetic condition that affects eye development and is most often caused by mutations that lead to the loss of one functional copy of the *PAX6* gene.^1^ A defining characteristic of aniridia is the lack of iris or the presence of an iris abnormality, but the most common ocular finding in aniridia is varying degrees of foveal hypoplasia.^2^^–^^4^ The *PAX6* gene plays a crucial role in normal fetal development of the eye and is a part of a transcriptional network that regulates photoreceptor specification and differentiation of both rod and cone photoreceptors, as well as retinal pigment epithelium (RPE) cells.^5^^–^^8^ In addition, expression of the gene continues through adulthood and is thought to play a role in maintenance of the retina, lens, and cornea throughout life.^9^

Mutations in *PAX6* or one of its regulatory regions result in an insufficient level of PAX6 protein and are associated with abnormal foveal formation,^10^^–^^13^ reduced cone density,^14^ and decreased cone-related visual function, such as visual acuity^2^ and color discrimination.^15^ Optical coherence tomography (OCT) has revealed significantly thinner outer retinal layers across the macula in persons with aniridia than in healthy eyes.^3^ Although many factors contribute to the decreased visual function in patients with aniridia, the level of visual acuity is related to the extent of foveal hypoplasia,^2^^,^^15^^,^^16^ with presumably further reduction due to the progressive anterior segment opacities.^4^ Assessment of retinal function with electroretinography (ERG) has demonstrated both abnormal cone and rod responses using full-field ERG and multifocal ERG in congenital aniridia.^17^^–^^19^ The dysfunction varies from nearly normal to severely affected, suggesting heterogeneity in the retinal function of persons who have a *PAX6* mutation. Previous studies of visual function in aniridia are mainly limited to the macular area; thus, the implication of these findings with regard to overall visual functions is not known.

Measurements of photoreceptors undergoing dark adaptation can yield important information about photoreceptor function,^20^ in particular that of the rods. The measure of dark adaptation quantifies the ability of the rod and cone photoreceptor functions to recover after exposure to light.^21^ It measures the lowest intensity of light required to detect a stimulus and is a basic measure of the integrity of the outer retina. Evaluating dark adaptation with the use of a dark adaptometer has not been investigated previously in aniridia, to the best of our knowledge. Because the *PAX6* gene is crucial for normal retinal development of numerous eye structures, including the neural retina and RPE, and because the above-mentioned ERG studies imply decreased rod function, it is reasonable to hypothesize that the dark-adaptation thresholds may be abnormal in patients with congenital aniridia when compared with normal age-matched controls.

The ability to adapt after a change from photopic to scotopic ambient lighting conditions is important in many situations in daily life. Individuals who have aniridia have either no iris or a very thinly pigmented iris,^22^ which means there is no or little modulation of light entering the retina, potentially prolonging the dark-adaptation process. Thus, knowledge about both rod and peripheral retinal function in aniridia will also be useful in developing guided treatment and advice. Therefore, the purpose of this study was to investigate dark-adapted cone and rod thresholds and their association with retinal structure as imaged by OCT in persons with congenital aniridia. The findings may provide valuable insights into the mechanism of retinal dysfunction.

## Methods

Written informed consent was obtained from all participants. The tenets of the Declaration of Helsinki were followed, and the study was approved by Regional Committee for Medical and Health Research Ethics (Southern Norway Regional Health Authority). This study was appended to a study of genotype and retinal phenotype in congenital aniridia.^3^ Twenty-seven of the original 37 participants with genetically confirmed *PAX6*-related aniridia (mutation in *PAX6* or one of its regulatory regions) and 38 age-matched healthy controls underwent dark adaptometry. As per the original study, a comprehensive eye examination of the anterior and posterior segment was performed, including best-corrected visual acuity, slit-lamp biomicroscopy, and grading of aniridia-associated keratopathy (AAK) and foveal hypoplasia, as well as *PAX6* genotyping.

The thickness of the inner and outer retinal layers was measured from spectral-domain optical coherence tomography images (SPECTRALIS OCT2 Module; Heidelberg Engineering, Heidelberg, Germany) of the macular region as described previously.^3^ In short, intraretinal reflectance peaks or boundaries corresponding to the inner limiting membrane, inner plexiform layer, outer plexiform layer, external limiting membrane, ellipsoid zone, interdigitation zone, and RPE–Bruch's membrane in the horizontal OCT B-scans taken through the foveal center were semi-automatically segmented using an active contour method.^23^ Previously reported foveal hypoplasia status, retinal layer thicknesses, and AAK were used in our analysis.^3^^,^^15^^,^^24^ AAK was graded from 0 to 3,^24^^,^^25^ where AAK grades 0 to 2 indicate that the central cornea remains unaffected. The type and severity of cataract were graded according to the Lens Opacities Classification System III.^26^ In the analysis, the sum of the grading of nuclear, cortical, and posterior subcapsular opacities was used to quantify lens opacity.

### Dark Adaptation

Rod- and cone-mediated dark adaptation was measured with a dark adaptometer (Roland Consult, Brandenburg, Germany) after a 5-minute exposure to bright white light (7000 cd/m^2^). Each participant had either their best eye (aniridia) or their dominant eye (healthy controls) assigned to undergo dark-adaptation testing. The pupil of each healthy participant was dilated with tropicamide 0.5% eye drops 20 minutes prior to examination. All of the patients with aniridia had full or partial iris hypoplasia so drops were not necessary. The participants were instructed to maintain fixation on a red fixation light and to press a response button when a flashing light stimulus became visible. A live view of the participant's test eye (using an infrared camera) was displayed to the operator, who verbally encouraged the participant to maintain fixation. An opaque patch occluded the fellow eye during testing. Two 2° circular light sources (with 527-nm and 625-nm wavelengths) were alternately tested at ≈20° eccentricity temporal of the fovea (corresponding to an area of high rod density)^27^^,^^28^ over a period of 35 minutes. For each light source, a staircase psychometric procedure tracked the participant's responses (the stimulus was dimmed by 6 dB if it was correctly detected or increased by 2 dB otherwise). When the experiment finished, the two test-response sequences could be used to estimate the detection thresholds of cones and rods, respectively.

### Analysis

**Figure 1.**
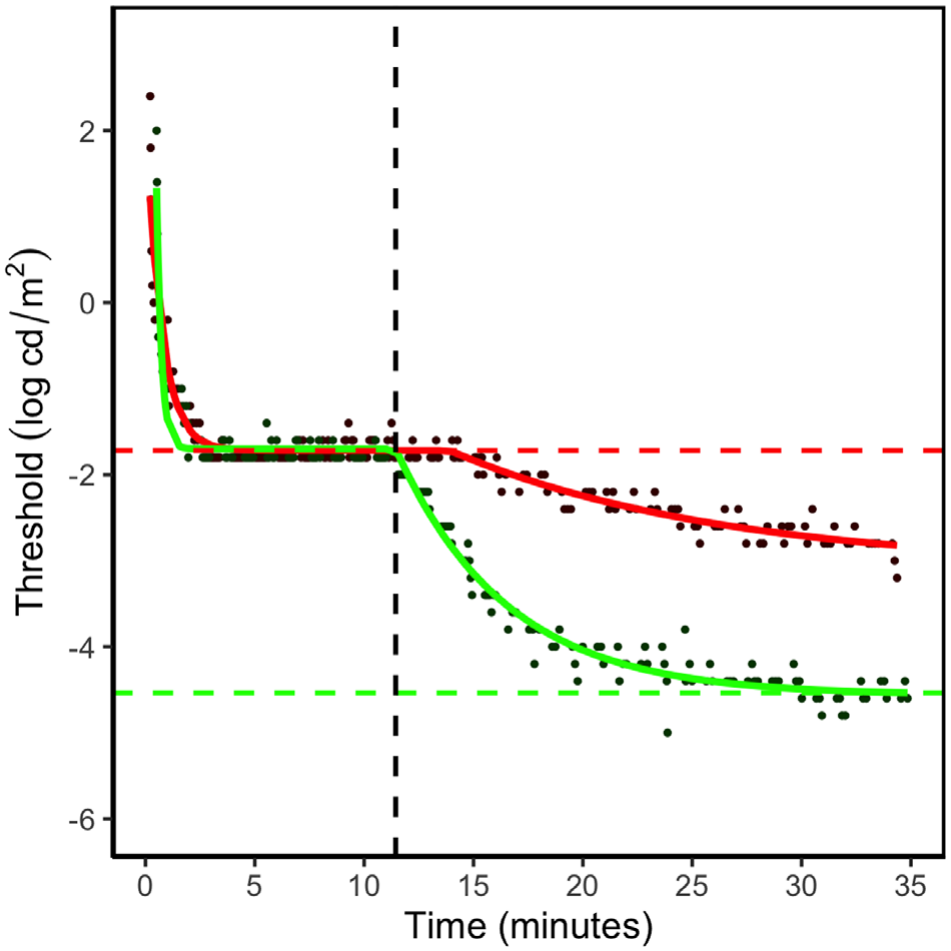
Example of dark-adaptation curves with the fitted two-stage exponential decay function for a healthy young adult following a 5-minute retinal bleach. The *red curve* represents the model fitted to the responses from the red-light (625 nm) stimulus and the *green curve* the model for the green-light (527 nm) stimulus. Cone threshold was derived from the *red curve* as illustrated here with the *red dashed line*. The final rod threshold was derived from the *green curve* (*green dashed line*). The time to rod–cone break represents the point in time during dark adaptation when the sensitivity of the rods first exceeds that of the cones (*vertical black dashed line*).

The two test-response sequences representing the participant's dark-adaptation profile (Fig. 1) were modeled as a two-stage exponential decay function,^29^^,^^30^ characterizing the initial cone-driven sensitivity followed by the emerging dominance of rod sensitivity:

$$\begin{array}{c} y=\left\{\begin{array}{cc} B_{c}+I_{c}e^{R_{c}t} & \mathrm{if}\;t<t_{b}\\ B_{c}+I_{c}e^{R_{c}t_{b}}-I_{r}e^{R_{r}t_{b}}+I_{r}e^{R_{r}t} & \mathrm{if}\;t\geq t_{b} \end{array}\right. \end{array}$$

where *y* is the detected light intensity in log units (log cd/m^2^), and *t* is the onset time of the test light (seconds since extinguishing of the bleach light). The parameters to be fitted were the magnitude (*I*), rate (*R*), and asymptote of decay (*B*) for cones and rods (subscripted *c* and *r*, respectively). The duration of the initial decay period (*t_b_*) represents the time point when rods became more sensitive than cones—the time to the rod–cone break (TRCB).

The dark-adaptation function was fitted independently to each of the red and green data using the Nelder–Mead minimization function, optim() in R (R Foundation for Statistical Computing, Vienna, Austria),^31^ to yield the cone and rod thresholds, respectively (Fig. 1). Each application of optim() used the same seed parameters.

### Statistical Analysis

The demographics and clinical characteristics of the participants were evaluated using traditional descriptive methods, such as mean, standard deviation (SD), 95% confidence intervals (CIs), median and range. The Welch two-independent-sample *t*-test was used to assess differences in age and dark-adaptation parameters between patients with aniridia and healthy controls. Mean differences in dark-adaptation parameters among different age groups, AAK grades, and intraocular lens status were examined using one-way analysis of variance or the Kruskal–Wallis rank-sum test with post hoc pairwise *t*-tests or Wilcoxon tests, respectively, with Bonferroni correction to account for multiple comparisons. Pearson's (*r*) or Spearman (*r_s_*) correlation coefficients were used to identify the correlation between dark-adaptation parameters and retinal structure, axial length, and sex. The choice of parametric or nonparametric tests was based on analysis of normality using the Shapiro–Wilk test, histograms, and Q–Q plots. Stepwise multivariate linear regression models were applied to assess the association between cone and rod dark adaptation thresholds (dependent variables) and retinal structure and anterior segment opacities. Models were compared using likelihood ratio tests. The significance level was set at α = 0.05. All analyses were performed in R 4.2.1.^31^ Relevant datasets and R scripts are available at the USN Research Data Archive (https://doi.org/10.23642/usn.21904242).

## Results

Dark adaptation was measured in 27 participants with congenital aniridia and in 38 age-matched healthy controls. One of the healthy controls was excluded from the analysis because of unreliable responses caused by a fault with the response box during the testing procedure. One patient with aniridia was excluded from the analysis due to subsequently being diagnosed with dry age-related macular degeneration (AMD). Thus, 26 participants with aniridia (nine males; 11–66 years old; mean, 33.8 ± 16.5 years) and 37 healthy controls (14 males; 10–74 years old; mean, 32.8 ± 18.6 years), *t*(57.7) = −0.24, *P* = 0.81, were included in the analysis. All healthy control eyes had a best-corrected visual acuity of 0.10 logMAR or better and no history of systemic or ocular disease. The retina was unremarkable on clinical assessment. The participants with aniridia had a median best-corrected visual acuity of 0.80 logMAR (range, 0.00–1.76). Ten participants had AAK grade ≤ 1, 11 had AAK grade 2 and five had AAK grade 3. OCT images of sufficient image quality were obtained from 20 of the participants with aniridia, of whom 95% (19/20) had some grade of foveal hypoplasia (1–4).

**Figure 2.**
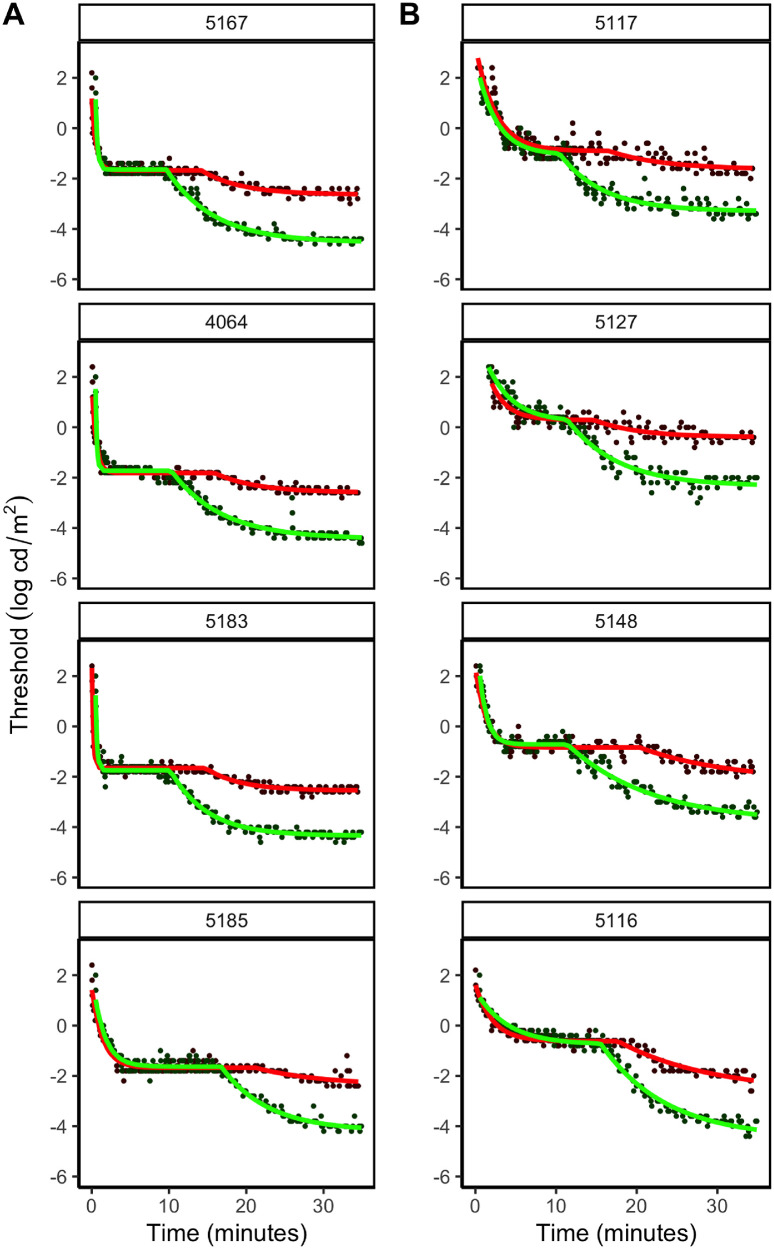
Dark-adaptation data and curves from the fitted two-stage exponential decay function overlaid for four age-matched healthy controls (**A**) and four representative participants with aniridia (**B**) from different age groups (from top to bottom: ∼20, ∼40, ∼50, and >65 years). When comparing the fitted curves in **A** with those in **B**, it is evident that both the rod and cone thresholds are more elevated in those with aniridia. It is also clear that the TRCB is increased in the oldest age group.

Figure 2 presents dark-adaptation curves with the fitted models plotted as thresholds in log cd/m^2^ versus time in minutes after bleach for four participants with aniridia from different age groups and four healthy age-matched controls. The fitted dark-adaptation functions for all participants are shown in Supplementary Figures S1 and S2. The TRCB varied between 6.5 and 19.7 minutes (mean, 11.4 ± 2.5) for healthy controls and were significantly longer for those 60 years and older compared with the younger age groups, *F*(2) = 8.55, *P* = 0.001. There was no significant association between the cone- or rod-mediated dark adaptation (cone threshold, TRCB, rod threshold) and sex or axial length.

**Figure 3.**
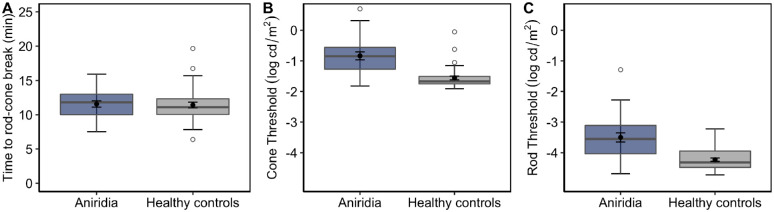
Boxplots showing the differences in dark-adaptation TRCB (**A**), cone threshold (**B**), and rod threshold (**C**) between the aniridia group (*blue boxes*) and the healthy control group (*gray* *boxes*). The *black dots with error bars* within the *boxes* represent the mean ± standard error.

Figure 3A shows that the TRCB in patients with aniridia (7.5–15.9 minutes; mean, 11.6 ± 2.3) was not different from that of the healthy controls, *t*(51) = −0.25, *P* = 0.80. In contrast, patients with aniridia showed larger variability and, on average, a significant elevation of both dark-adapted cone thresholds (−0.84 ± 0.66 and −1.56 ± 0.36 log cd/m^2^ for aniridia and healthy controls, respectively), *t*(35.6) = −5.1, *P* < 0.0001 (Fig. 3B), and rod thresholds compared with healthy age-matched controls (−3.50 ± 0.76 and −4.23 ± 0.34 log cd/m^2^ for aniridia and healthy controls, respectively), *t*(32.2) = −4.6, *P* < 0.0001 (Fig. 3C). However, some patients with aniridia retained thresholds within the normal range.

There was a statistically significant difference in cone thresholds between different grades of AAK, *H*(2) = 7.02, *P* = 0.03, where those with grade 3 AAK had significantly higher thresholds than those with grade 0 or 1 AAK (*P* = 0.014). Rod thresholds also tended to be higher with more severe grades of AAK, *H*(2) = 5.44, *P* = 0.07, but not significantly. We did not find a significant correlation between lens opacities and either cone threshold (*r_s_* = 0.43, *P* = 0.13) or rod threshold (*r_s_* = 0.39, *P* = 0.17) in the 14 patients with phakic aniridia. There was also no difference in thresholds among phakic, aphakic, and pseudophakic participants, *H*(2) = 1.01, *P* = 0.69 and *H*(2) = 1.20, *P* = 0.55 for dark-adapted cone and rod thresholds, respectively.

Measurements of the retinal layers were available in 20 of the patients (and all of the healthy controls). Five patients with stage 3 AAK, where the central cornea is affected, were excluded because of poor image quality. Thus, all of those included in the analysis of the relationship between the retinal structure and dark adaptation parameters had AAK grade ≤ 2.

**Figure 4.**
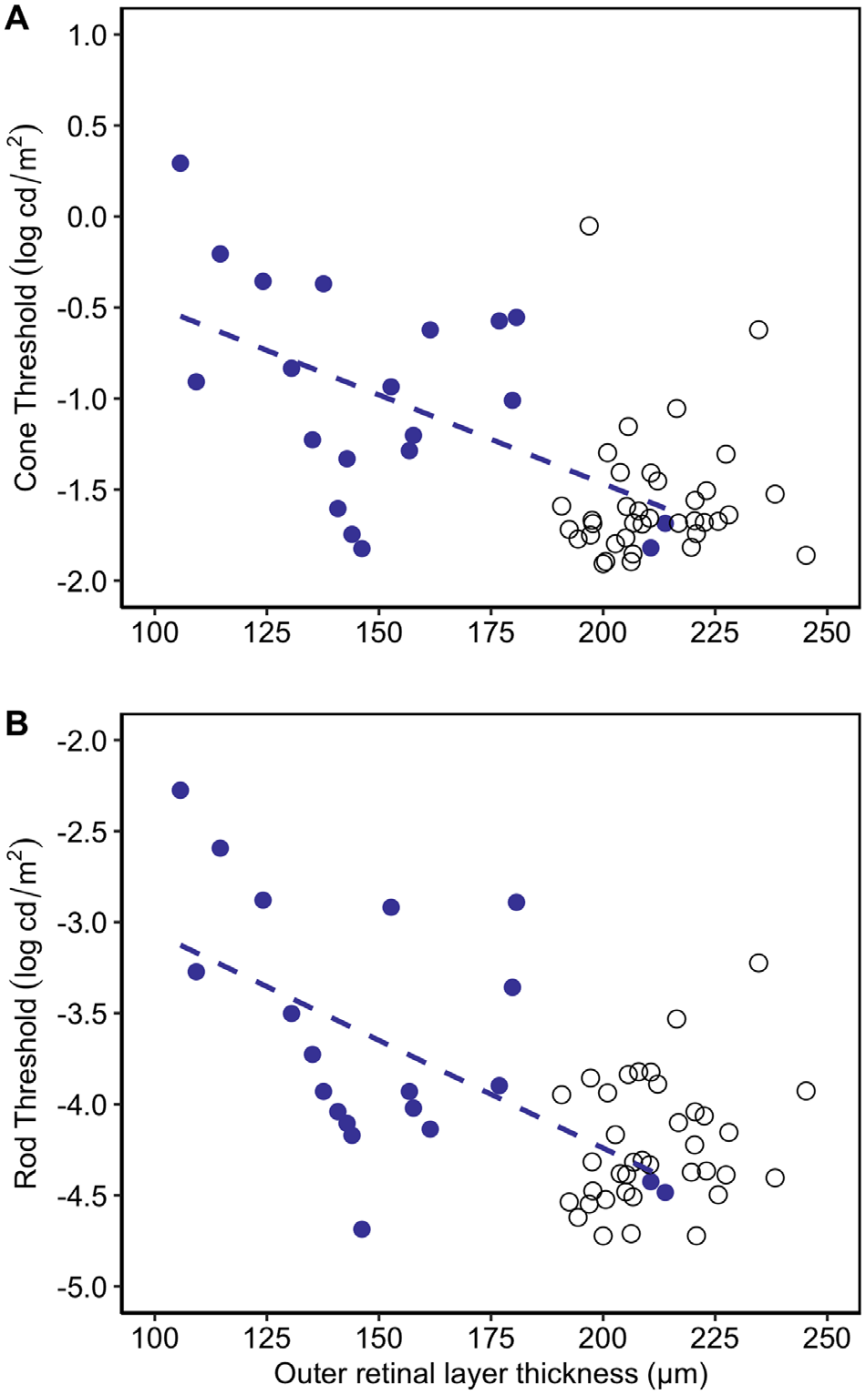
Cone thresholds (**A**) and rod thresholds (**B**) plotted as a function of central outer retinal layer thickness. Participants with aniridia are represented by the *blue filled circles* and the *dashed regression line*. Data from the healthy controls (*open circles*) are shown for comparison.

In aniridia, there were no significant relationships between total retinal thickness or outer retinal thickness in the perifovea (1.5–3.0 mm retinal eccentricity) for either dark-adapted cone thresholds (*r* = −0.27, *P* = 0.26 and *r* = −0.24, *P* = 0.31, respectively) or dark-adapted rod thresholds (*r* = −0.38, *P* = 0.097 and *r* = −0.29, *P* = 0.22, respectively), although there was an association between thinner perifoveal retinal thickness and higher rod thresholds when including age as a factor (*P* = 0.022). In contrast, Figure 4 shows that the central outer retinal layer thickness correlated significantly in aniridia with both cone thresholds (*r* = −0.49; 95% CI, −0.77 to −0.06; *P* = 0.027) and rod thresholds (*r* = −0.53; 95% CI, −0.79 to −0.11; *P* = 0.017).

Based on these analyses, central outer retinal layer thickness, age, and AAK grade were included as independent variables in the multivariate regression model for prediction of dark-adapted cone thresholds. Perifoveal retinal thickness was included as an additional independent variable for prediction of dark-adapted rod thresholds. The best model prediction for both cone and rod thresholds in aniridia was when only age and central outer retinal layer thickness were included in the model, *r*^2^ = 0.38, *F*(2, 17) = 5.29, *P* = 0.016 and *r*^2^ = 0.45, *F*(2, 17) = 7.16, *P* = 0.006 for cone and rod threshold, respectively. Central outer retinal layer thickness significantly predicted the cone threshold (β = −0.011, *P* = 0.009) and rod threshold (β = −0.014, *P* = 0.004), as did age for the rod threshold (β = 0.019, *P* = 0.030), but was only near significance for cone threshold (β = 0.015, *P* = 0.06).

## Discussion

This study confirms our hypothesis that participants with aniridia have significantly lower sensitivity (higher thresholds) for both cone and rod function after dark adaptation than healthy age-matched participants. The reduced cone and rod sensitivity seems to be related to a combination of parameters, suggesting that retinal functional changes in *PAX6*-related aniridia are not limited to the central macular area but also include cone and rod function in the mid periphery. The association between dark-adaption thresholds and foveal thickness of the outer retinal layers may reflect that there is a link between foveal development^32^^–^^34^ and peripheral parts of the retina. This is supported by a previously reported correlation between foveal outer retinal layer thickness and thickness of the perifoveal inner and outer retinal layers and total retinal thickness in aniridia.^3^

Although scotopic vision is an important part of a person's functional vision, there has been little focus on rod function in congenital aniridia except the reported abnormal ERGs in the majority of the patients with aniridia, including abnormal dark-adapted rod responses.^17^^,^^19^ This is in line with our study, which suggests that the rod system in aniridia is less sensitive than in healthy age-matched controls, in terms of elevated dark-adaptation thresholds. The data from both the healthy controls and those with aniridia showed similar shapes (classical biphasic) of the dark-adaptation curves. This suggests a similar rate of sensitivity recovery, although the sensitivity was reduced in patients with aniridia compared with that of normal observers. The TRCB was increased in the oldest age group, which is consistent with findings reported by previous studies.^35^

The time taken to adapt to scotopic conditions (TRCB) was similar for patients with aniridia and normal observers. In functional terms, this implies that the typical iris abnormalities observed in aniridia do not appear to influence the onset of dark adaptation. However, we cannot rule out that the iris abnormalities alone increase incidences of photopigment bleaching^21^ and thus prolong dark adaptation in daily life. Furthermore, it indicates that the reduced photoreceptor sensitivity (increased thresholds) is independent of the molecular mechanisms involved in dark adaptation, as the onset of dark adaptation seems unaffected. This raises the question of why dark-adapted sensitivity was reduced. We surmise it could be due to an alteration in either the size and number of cone and rod photoreceptors or the neural pathways that subserve cone and rod function, or both. An altered distribution or number of photoreceptors is likely. In support of this, a lower than normal cone density within the macula has been reported in aniridia using adaptive optics scanning light ophthalmoscopy.^14^ It is not known how the rod density is affected, but it is likely to be altered because of the critical role of *PAX6* in early embryonic retinal development, where it controls the development of individual retinal cell types, including cone and rod photoreceptors, through several mechanisms.^7^^,^^12^^,^^36^ It is possible that a low redundancy of photoreceptor cells can make the retina more vulnerable to other ocular pathology that may affect retinal sensitivity. The second aspect is that the loss of one functional copy of *PAX6* or the reduced number of photoreceptors itself also affects the number and distribution of the postreceptoral neurons (for example, amacrine and retinal ganglion cells, which are essential for signaling in the dark-adapted retina).^37^^–^^40^ An alteration of the postreceptoral retinal cell density is supported by the thinner parafoveal and perifoveal inner retinal layers seen in aniridia.^3^

Vision loss in aniridia is multifactorial, thus it is challenging to predict visual function from retinal structure alone. Central ocular media opacities caused by AAK or cataract could affect how much light enters the eye and, thus, the amount of bleaching and/or sensitivity to detect light. However, the participants with an AAK grade that affected the central part of the cornea (AAK grade > 2) were excluded from the analysis, as they had no gradable OCT images. There was no association between corneal opacities and cone or rod dark-adaptation thresholds when the AAK grade was ≤2, nor between lens opacities and dark-adaptation thresholds. Thus, it is unlikely that ocular media opacities affected the dark-adapted cone and rod sensitivities measured in this study.

### Strengths and Limitations

The stimulus eccentricity (20°) was chosen to test an area on the retina with high rod density,^28^^,^^41^ as rod function was an important focus in this study. One potential limitation was that the retinal layer thicknesses were measured in the fovea, parafovea, and perifovea and not at 20° eccentricity. A direct correlation between structure and function in a given area was therefore not possible. However, we have demonstrated that there is an association between extramacular retinal function and structural changes within the macula that may shed further light on the underlying mechanisms of retinal development in aniridia.

Another possible limitation concerns fixation stability. Although the measures of dark adaptation were essentially confined to a single retinal location (to eliminate the effect of stimulus location on the relative difference between the participants), the nystagmus commonly found in patients with aniridia was significant and likely impacted upon fixation stability. Furthermore, it is uncertain what would be the most ideal location of the stimulus to measure dark adaptation in this patient group. It is possible that moving the stimulus to a more central location may show greater differences, particularly in cone sensitivity, because the density of foveal cones is greatly reduced compared to normal cone density.^3^ Consequently, we can speculate whether the rod mosaic may also be more affected in a more central location and show a larger relative difference in dark-adaptation thresholds between those with aniridia and normal observers.

An important strength of the test procedure was that it allowed for assessment of the full dark-adaptation function, including both cone and rod dark adaptation and final thresholds. Such assessment is relevant to characterizing scotopic visual function in persons with congenital aniridia and thus strengthen clinical management by providing better, well-founded, individual alternatives for facilitation and vision rehabilitation. The method does, however, suffer from being time consuming for a clinical procedure and can be biased by patient fatigue. A shorter testing time and less intense adaptation light could allow scotopic function to be more readily and efficiently probed in aniridia, in addition to allowing more frequent, less distressing testing of patients. The measurement will then be limited to include only a part of the dark-adaptation curve. Alternatively, rod intercept time (time taken to detect a stimulus with a predefined criterion threshold) is a sensitive method for detecting early AMD, even before structural changes are visible,^42^ and may find a role in management of aniridia. However, further investigation is warranted.

In conclusion, aniridia participants have dark-adaptation curves with thresholds that are elevated compared with those of age-matched healthy controls. Our results indicate that both rod and cone functions are affected and suggests that retinal changes in *PAX6*-related aniridia are not confined to the central macula. These findings provide new insight into visual function in aniridia; however, the association between arrested foveal development and more peripheral retinal changes requires further studies.

## Supplementary Material

Supplement 1

Supplement 2
