# Supplementary material for: Rod and Cone Dark Adaptation in Congenital Aniridia and Its Association With Retinal Structure
Source: Invest Ophthalmol Vis Sci. 2023 Apr 17;64(4):18. doi: 10.1167/iovs.64.4.18 (PMC10120381; doi:10.1167/iovs.64.4.18)
Supplement: Supplement 1 [file iovs-64-4-18_s001.pdf]

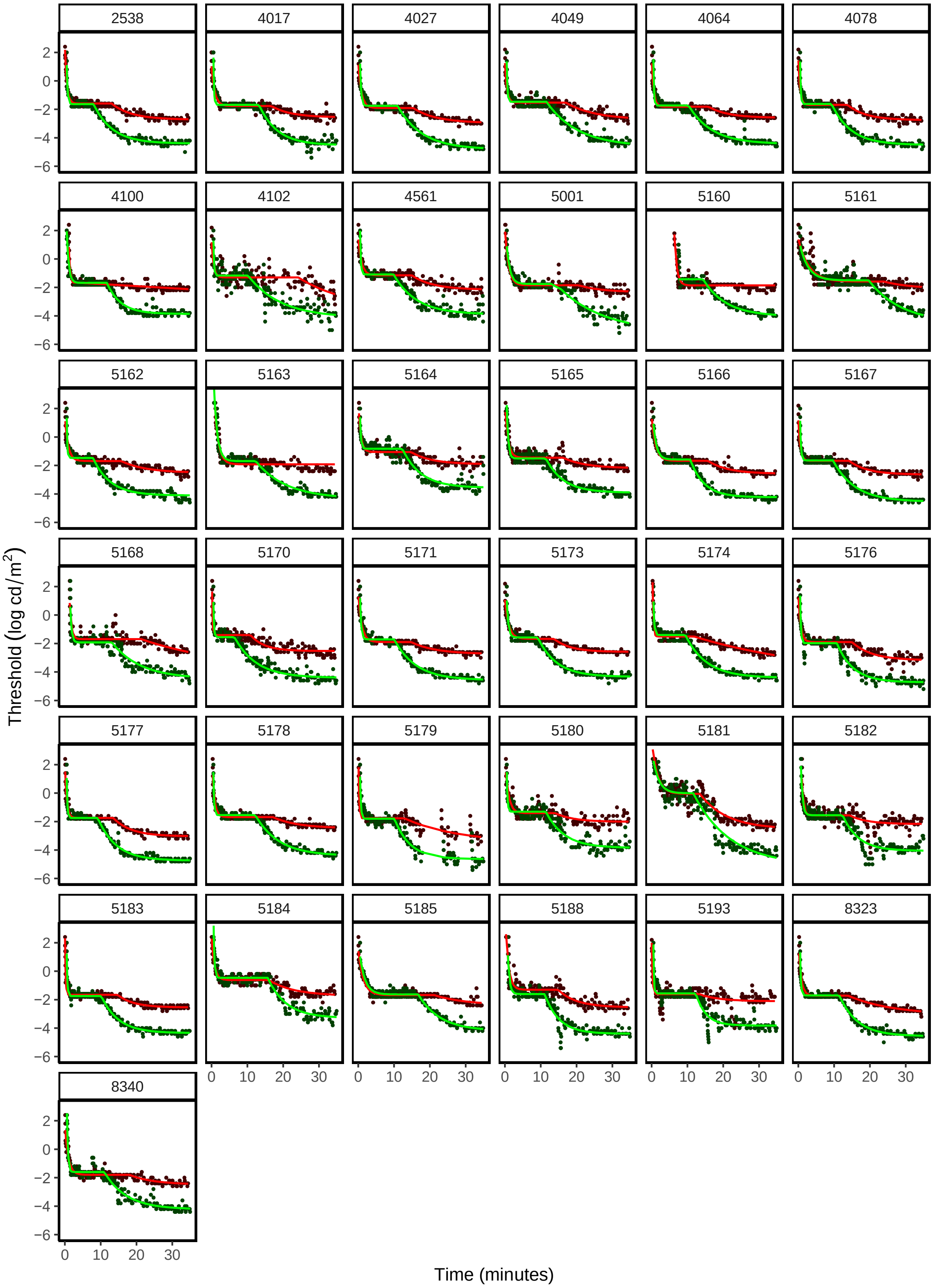

Supplementary Figure S1. Dark-adaptation data and curves from the fitted two-stage exponential decay function overlaid, for all the 37 age-matched healthy controls.
